# Pheromones of three ambrosia beetles in the *Euwallacea fornicatus* species complex: ratios and preferences

**DOI:** 10.7717/peerj.3957

**Published:** 2017-10-23

**Authors:** Miriam F. Cooperband, Allard A. Cossé, Tappey H. Jones, Daniel Carrillo, Kaitlin Cleary, Isaiah Canlas, Richard Stouthamer

**Affiliations:** 1 Otis Laboratory, APHIS-PPQ-S&T, United States Department of Agriculture, Buzzards Bay, MA, United States of America; 2 Former address: Agricultural Research Service—NCAUR, United States Department of Agriculture, Peoria, IL, United States of America; 3 Department of Chemistry, Virginia Military Institute, Lexington, VA, United States of America; 4 Tropical Research and Education Center, University of Florida, Homestead, FL, United States of America; 5 Department of Entomology, University of California, Riverside, Riverside, CA, United States of America

**Keywords:** Pheromone, Polyphagous shot hole borer, Tea shot hole borer, Repellent, Attractant, Kuroshio shot hole borer, Quercivorol, Kairomone, Chemical ecology

## Abstract

Three cryptic species in the * Euwallacea fornicatus* species complex were reared in laboratory colonies and investigated for the presence of pheromones. Collections of volatiles from combinations of diet, fungus, beetles, and galleries from polyphagous shot hole borer (*Euwallacea* sp. #1) revealed the presence of 2-heneicosanone and 2-tricosanone only in the presence of beetles, regardless of sex. Subsequent examination of volatiles from the other two species, tea shot hole borer (*Euwallacea* sp. #2) and Kuroshio shot hole borer (*Euwallacea* sp. #5), revealed these two ketones were present in all three species but in different ratios. In dual choice olfactometer behavioral bioassays, mature mated females were strongly attracted to a synthetic binary blend of ketones matching their own natural ratios. However, females in each species were repelled by ketone blends in ratios corresponding to the other two species. Males of each species responded similarly to females when presented with ratios matching their own or the other two species. The presence of these compounds in the three beetle species, in ratios unique to each species, and their strong species-specific attraction and repellency, suggests they are pheromones. The ecological function of these pheromones is discussed. In addition to the pheromones, the previously known attractant (1*S*,4*R*)-*p*-menth-2-en-1-ol (also known as quercivorol) was discovered in the presence of the fungal symbionts, but not in association with the beetles. Quercivorol was tested in a dual-choice olfactometer and was strongly attractive to all three species. This evidence suggests quercivorol functions as a kairomone for members of the * E. fornicatus* species complex, likely produced by the symbiotic fungi.

## Introduction

Until several decades ago, ambrosia beetles were not considered economically or ecologically important pests of living trees because the vast majority of them cultivate their ambrosia fungus within already dead or dying trees and other woody host plants and function ecologically as decomposers (Batra, 1963). However, recently it has been realized that some ambrosia beetles are capable of attacking healthy trees where their ambrosia fungus functions as a plant pathogen, infecting trees and causing branch dieback, wilt, or tree mortality (Kühnholz, Borden & Uzunovic, 2001; Hulcr et al., 2017). With the sharp increase of global trade in recent years, we have also seen an increase of invasive ambrosia beetles capable of causing major economic and ecological damage, and severely threatening native forest ecosystems (Marini et al., 2011). Such is the case with members of the *Euwallacea fornicatus* species complex (Coleoptera: Curculionidae: Scolytinae).

Independent studies have concluded that populations of beetles morphologically identified as *E. fornicatus,* that stem from four separate invasions in the United States (Hawaii, Florida, and two in southern California), are composed of three genetically distinct, cryptic species of ambrosia beetles in what is now recognized as the *E. fornicatus* species complex (Eskalen & Stouthamer, 2012; Eskalen et al., 2013; O’Donnell et al., 2015; Stouthamer et al., 2017). All three species morphologically resemble *E. fornicatus*, but they are genetically different enough to be considered different species, and carry different species of fungal symbionts in the genus *Fusarium* (O’Donnell et al., 2015; Carrillo et al., 2016). They have yet to receive unique scientific names, but they are commonly referred to as: the polyphagous shot hole borer (PSHB) (*Euwallacea* sp. #1), which was first detected in Los Angeles County, CA in 2003 (Eskalen et al., 2012; Eskalen et al., 2013); the tea shot hole borer *sensu lato* (TSHB) (*Euwallacea* sp. #2), which was first detected in Hawaii in 1910 (Schedl, 1941) and more recently in Miami-Dade County, FL in 2002 (Rabaglia et al., 2008); and the Kuroshio shot hole borer (KSHB) (*Euwallacea* sp. #5), which was first detected in San Diego County, CA in November 2013 (Eskalen et al., 2013; O’Donnell et al., 2015; Carrillo et al., 2016; Boland, 2016; Stouthamer et al., 2017; Dodge et al., 2017). Each of these three beetle species carry different species of symbiotic *Fusarium* in their mandibular mycangia (O’Donnell et al., 2015; Carrillo et al., 2016), and the inability of PSHB and TSHB larvae to survive when fed *Fusarium* from the other species suggests that isolation between species also takes place in their obligatory feeding requirements for their associated *Fusarium* species as well (Freeman et al., 2013a). Differences were also found between the cuticular hydrocarbon profiles of PSHB and TSHB which could potentially assist in species diagnostics since they are morphometrically indistinguishable (Chen et al., 2016). These three cryptic species are similar in their polyphagous nature, in that they can attack and spread their *Fusarium* symbiont to hundreds of tree species in numerous families (Danthanarayana, 1968; Eskalen et al., 2013). According to Eskalen et al. (2013) and Eskalen (2016), there are now 49 known reproductive hosts of PSHB, and 15 known for KSHB (see also Boland, 2016). These lists continue to expand rapidly as research on these species continues to unveil their numerous reproductive hosts. They threaten numerous native tree species in California. For instance, in riparian forests in San Diego county along the border with Mexico, KSHB has attacked and severely damaged the majority of the three dominant native willow species, *Salix lasiolepis*, *S. gooddingii,* and *S. laevigata*, which profoundly affects the entire ecosystem (Boland, 2016). California sycamore, *Platanus racemosa,* is another dominant native tree species that is susceptible to mass attack and killed by PSHB and KSHB (Coleman, Eskalen & Stouthamer, 2013; Boland, 2016). Avocado is now threatened in California and Florida, and more than one quarter of all street trees in southern California are reproductive hosts susceptible to attack (Lesser, 1996; Eskalen & Stouthamer, 2012; Mendel et al., 2012; Freeman et al., 2013b; Eskalen et al., 2013; Carrillo et al., 2016; Cooperband et al., 2016; Kendra et al., 2017; Stouthamer et al., 2017).

These three cryptic species of beetles collectively bring with them at least five species of phytopathogenic *Fusarium* ambrosia which they cultivate, and upon which they feed and develop inside galleries in trees and woody plants (O’Donnell et al., 2015; Carrillo et al., 2016). Infection of trees with these fungi cause the disease known as Fusarium dieback. Additional fungi, *Graphium euwallaceae*, *Paracremonium pebium*, and *Acremonium* sp. were found in the heads of beetles from California and Florida (Lynch et al., 2016; Carrillo et al., 2016). The fungal symbionts help the beetles overcome defenses of a seemingly healthy tree by blocking the vascular tissues of the tree, subsequently lead to staining, branch dieback, and large scale tree mortality (Eskalen et al., 2013; Lynch et al., 2016). Interestingly, a positive association has been seen between water abundance and beetle infestation rate (Boland, 2016).

Mating typically occurs between haploid brothers and diploid sisters in their natal galleries prior to female dispersal (Cooperband et al., 2016). A female that has not found a mate may initiate a new colony by producing haploid male offspring through parthenogenesis, mating with a son, then producing female offspring (Cooperband et al., 2016). Therefore, inbreeding is the rule, and outbreeding depression is likely (Peer & Taborsky, 2005). A crossing study conducted between PSHB and TSHB revealed that when forced to interbreed, most crosses failed, but a small amount of hybridization resulted in low fitness or reproductive compatibility between the two species (Cooperband et al., 2015). Results were similar when attempting to cross PSHB and KSHB, demonstrating that there is reproductive isolation between the species (Cooperband et al., 2017).

The three beetle taxa in the *E. fornicatus* species complex originate in southeast Asia, and there are regions where they occur in sympatry (Stouthamer et al., 2017). The most genetically diverse populations of TSHB were in Thailand, PSHB in Vietnam and Taiwan, and KSHB in Taiwan, suggesting their possible evolutionary origins. However, all three species were found in Taiwan, PSHB and KSHB were both found in Okinawa, and PSHB and TSHB were both found in Thailand (Stouthamer et al., 2017). Although geographical barriers play a role in genetic isolation between species, with overlapping host tree and geographical ranges, other character displacements may also play a role in the genetic isolation between the three species.

With the need for improved detection tools soon after the invasion of PSHB in southern California, the initial goal of this study was to investigate the possible presence of a pheromone. As studies began to emerge establishing that three distinct cryptic species occur in the US, the scope of this study expanded to encompass all three species. The goal, if pheromones were found, was to identify and quantify them, and demonstrate their behavioral function. Because of the potentially confounding presence of behaviorally activevolatiles from the host plant and the symbionts, experiments were designed to isolate volatiles originating from beetles while controlling for those that originated from their fungal symbionts or host plant.

## Materials and Methods

### Insects

Initial exploratory volatile collections focused only on beetles from the population of PSHB (*E.* sp. #1) collected in Altadena, in Los Angeles County in southern California, which has been maintained in colony in the insect containment facility of the Otis Laboratory since August, 2013 (USDA permit P526P-13-01673) (Cooperband et al., 2016).

Subsequent volatile collections and extracts to compare the three members of the species complex involved PSHB as well as TSHB (*E.* sp. #2) isolated from Miami-Dade County in Florida and reared in a laboratory colony since early 2014, and KSHB (*E*. sp. #5) which was isolated from San Diego County, CA and kept in a laboratory colony since the end of 2014. Rearing took place under LD 16: 8 h photocycle at 24 °C, using protocols described in detail in Cooperband et al. (2016). Briefly, sib-mated females were placed individually into 50 ml polyethylene centrifuge tubes (Fisher Scientific, Waltham, MA) containing 15 ml of artificial diet. Diet was based on sawdust from either boxelder (for PSHB) or avocado (for TSHB and KSHB), corresponding to host tree from which they were originally collected. Initially each foundress excavated into the diet, seeding it with *Fusarium* fungus from her mycangia (Freeman et al., 2013a; O’Donnell et al., 2015), and forming galleries lined with *Fusarium* which would be fed upon by her and her offspring over the next 5–8 weeks. During that time the 15 ml diet plug became completely permeated with the fungus. On average, a typical foundress produced between 25 to 35 females and one to three male offspring in 5–8 weeks (Cooperband et al., 2016). The three species were reared separately, and to avoid contamination between colonies they were kept in separate triple-nested containers which were never opened at the same time. Containers and work areas were wiped with a 10% solution of commercial bleach before and after use. Beetles and *Fusarium* species were genetically confirmed to match those described by O’Donnell et al. (2015). Beetles were identified using sequences of COI (Cooperband et al., 2016) whereas the fungi were identified using sequences of the rDNA internal transcribed spacer region (ITS rDNA) and 28S rDNA D1/D2 domains (see O’Donnell et al., 2015 for primers and reaction conditions).

### Volatile collections for qualitative comparisons with PSHB

The first phase involved exploration for a pheromone by collecting volatiles from sources with and without PSHB beetles and comparing volatile profiles for qualitative differences. To maximize this phase, we employed several approaches to collect volatiles: solid phase micro-extraction (SPME) fibers, aerations, and solvent extracts or rinses on subjects with setups described below.

All SPME sampling utilized 100-µm polydimethylsiloxane coated fibers (Supelco, Bellefonte, PA). SPME fibers were exposed either: (1) in the headspace of a closed rearing tube or jar containing the volatile source, (2) inside a Pasteur pipette containing the volatile source, (3) inside the galleries of beetle colonies established in artificial diet, or (4) swiping or briefly touching the volatile source with the SPME fiber. Colonies were on average 47 d old when used and SPME fibers were exposed inside Pasteur pipettes for an average of 12 h. To sample the volatiles inside a gallery, the diet plug was tapped out of the rearing tube containing a mature beetle colony, and the bottom of the plug was chipped away incrementally until a gallery was revealed. A SPME fiber was inserted directly into the gallery and held in place for 2 min on average. After exposure, the diet plug was dissected, and the number and sex of beetles within that colony was quantified. In some cases, the foundress had died and no beetles were in the galleries, and these were re-categorized as part of the “diet + fungus” treatment (described below). To sample volatiles using a Pasteur pipette, approximately 150 mg of the source material or a known number of beetles was placed inside a glass Pasteur pipette, with the larger opening covered with aluminum foil, and the SPME fiber inserted and exposed through the smaller opening for on average 105 min. Alternatively, SPME fiber exposures in other containers such as the headspace inside a rearing tube lasted on average 272 min, and exposure inside galleries was on average 1 min.

Aerations in this phase were conducted by passing odor-laden air through volatile traps containing approximately 20 mg of either activated charcoal (50–200 mesh; Fisher Scientific, Waltham, MA, USA) or Hayesep Q (80–100 mesh; Hayes Separations, Inc., Bandera, TX, USA) packed between two small plugs of glass wool inside a Pasteur pipette. Air passed through an activated charcoal in-line air filter (Analytical Research Systems, Inc., Gainesville, FL, USA) at 0.2 L/min, then into a 50 ml rearing tube, 20 ml vial, or 0.24 L jar containing the odor source, and then exited the container through the volatile collection trap. Volatile samples were eluted with approximately 1 ml of hexane through the trap into a collection vial.

Extracts in this phase were made by placing the beetles into a 2 ml autosampler vial containing just enough hexane to cover them, and allowing them to soak for a period of time, from 30 min to several days. To make a rinse, live beetles were removed from their galleries, placed into a Pasteur pipette, and approximately 1 ml of hexane was dispensed into the pipette rinsing over the beetles and collecting in an autosampler vial. One rinse was made by dispensing the hexane directly into a gallery of a live colony of beetles, and immediately recovering the hexane with a Pasteur pipette.

For qualitative comparisons, odor sources were categorized into six treatments as follows:

(1) “Control” consisted of a clean container such as an empty Pasteur pipette or rearing tube.
(2) “Diet” consisted of sterile diet that had never been in contact with beetles or their fungal symbionts.
(3) “Diet + Fungus” consisted of the *Fusarium*-infested diet from the middle of a diet plug from a rearing tube, taken from an area that did not contain any galleries or beetles. One exception in which the gallery was included in this category occurred when a gallery was sampled from a rearing tube, but after dissection it was found that there were no living beetles in that tube.
(4) “Diet + Fungus + Beetles” consisted of non-gallery *Fusarium-* infested diet from rearing tubes as in “Diet + Fungus” above, but with beetles added (either male or female or both). This category mostly consisted of SPME samples taken from material placed inside of a pipette. However, this category also included head space volatile collections of rearing tubes containing complete colonies.
(5) “Gallery” refers to volatile samples that were taken from the gallery itself, in which live beetles were present. These were accomplished either by inserting a SPME fiber directly inside an inner gallery near the bottom of the diet tube, or by removing a section of inner gallery and placing it inside a Pasteur pipette, and then inserting the SPME fiber into the pipette. Also included in this treatment was the single hexane rinse of a gallery, described above. Each rearing tube from which a gallery was sampled was dissected and the number of males and females living in that tube was recorded and attributed to that gallery sample. Therefore, diet and fungus and beetles were all components of galleries.
(6) “Beetles” consisted of only beetles. They were removed from their galleries in a rearing tube and immediately sampled for volatiles in the absence of their diet and fungus rearing media. The beetle category was later broken down into three subcategories, male, female, or male + female, and compared to each other and to non-beetle samples.

### Volatile collections for qualitative comparisons with TSHB

While conducting the above sampling with PSHB, the first TSHB colony in a diet tube arrived from Florida. The TSHB colony had been initiated by a single field-collected foundress, surface sterilized in 70% ethanol for 10 s prior to introduction onto the diet. After developing for nine weeks it was used to test for volatiles. The colony was dissected and found to be densely populated with 69 adult females and five adult males. At this advanced colony age, all 15 ml of diet in the tube contained the *Fusarium*. Four volatile sources were selected from within the rearing tube and sampled with SPME fibers: (1) approximately 150 mg of diet and fungus from a solid area without beetles or galleries was placed inside a Pasteur pipette, (2) approximately 150 mg of the same diet and fungus from an area without beetles or galleries was placed in a second Pasteur pipette, and five adult male beetles were added, (3) 46 female beetles were placed in a sterile 120 ml specimen jar, and (4) the space inside beetle galleries. SPME fibers were exposed for 10, 10, 1, and 2 min to these four treatments, respectively.

### Exploratory sample analysis

Samples were analyzed by injection into an Agilent 7890B gas chromatograph coupled with a 5977A mass-selective detector (GC-MS) (Agilent Technologies, Inc., Santa Clara, CA, USA). The GC was equipped with an HP-5MS column (30 m × 0.25 mm I.D. × 0.25 µm film thickness; Agilent Technologies, Inc., Santa Clara, CA, USA). The column effluent was split in half by a Gerstel uFlow Manager (Gerstel Inc., Linthicum, MD, USA), such that half the effluent was directed into the MS and half to another detector that was not used in this study. Helium was used as the carrier gas (constant pressure 13.8 psi) and samples were injected in splitless mode. The GC injector was held at 250 °C, and the column starting temperature was 50 °C, held for 0.75 min, then ramped at 10 °C/min to 250 °C and held for 25 min. Initial GC-MS identifications were made by using libraries (Wiley and NIST), and subsequent verification of compounds compared Kovat’s indices, mass spectra, and retention times with those of synthetic standards (see Chemical synthesis section below). GC-MS results from different treatments were compared to look for compounds unique to beetles.

### Whole beetle extracts to compare pheromone component ratios among species

Beetles from each of the three species were gathered from galleries and groups of nine to 31 mature females (each group harvested from a different diet tube), and 3 to 10 males (combined from multiple tubes) were extracted in pentane for 30 min, after which a known amount of 2-tridecanone was added as an internal standard to allow for accurate quantification. Samples were analyzed on an Agilent 7890 GC equipped with a flame ionization detector (FID), using the above mentioned GC column and GC run settings.

### Bioassay design

Rearing tubes that were 5–11 weeks old were harvested and the mature adult females were placed in a holding jar with a piece of filter paper and allowed to acclimate for at least 1 h prior to use in behavioral bioassays.

Custom bioassay “Y-plates” designed by M Cooperband and manufactured by Applied Plastics Technology, Inc. (Bristol, RI, USA) were used to conduct dual choice behavioral bioassays within the insect containment facility at the Otis Laboratory. Each Y-plate consisted of a block of solid Teflon (16.5 long × 12.7 wide × 1.3 cm high) from which a channel was cut in the shape of a Y. A transparent sheet of acetate was placed against the top and bottom of the bioassay plate and sealed in place with a thin film of electrode gel, so air entering the two upper arms could only exit through a 1.905 cm diam. hole at the end of the stem. An oiless air compressor provided air flow through the apparatus via a regulator, activated charcoal filtration, Teflon tubing, humidifier, and a flow meter set to 0.6–0.7 L/min. Air was directed through a Y-splitter which delivered even flow to both upwind arms of the Y-plate. Visualization of the plume using smoke revealed that the plumes entering the two arms of the Y remained separate until they were nearly at the end of the stem. A hotwire anemometer placed at the downwind end of the Y was used to measure the wind speed, which was in the range of 30–35 cm/s.

### Chemical synthesis

The compounds 2-heneicosanone (2-21:Kt) and 2-tricosanone (2-23:Kt) were prepared from 1-bromooctadecane and 1-bromoeicosane respectively using previously described methodology (Mason et al., 1990). The resulting ketoesters were saponified, and after hydrolysis, provided the appropriate ketones. These ketones were recrystallized from heptane to provide more than one gram of each as a crystalline solid. The resulting material was 96.5 and 95.8% pure, respectively. Quercivorol, (1*S*,4*R*)-*p*-menth-2-en-1-ol, was prepared by following Mori (2006) and was found to be 99% pure (GC-FID), and the enantiomeric purity was determined to be 98% ee (chiral GC-MS).

### Lures for behavioral bioassays

Red rubber septa were extracted and loaded according to Zilkowski et al. (2006). The two synthetic ketones, 2-21:Kt and 2-23:Kt, were weighed and combined in hexane to produce stock solutions of each of the three ratios 45:55, 68:32, and 87:13. They were then serially diluted such that 100 ul contained either 0.25, 2.5, or 25 ug of the total combined ketones at the three different ratios. Septa loaded with the different doses and ratios of the two ketones were sliced into eighths, which were used in behavioral bioassays. Thus septum eighths used in the dose response assays contained approximately 31, 313, or 3,125 ng of the two ketones combined, at a ratio of 45:55. Septum eighths used to compare attraction for all three species contained 313 ng of the two ketones combined, at either 45:55, 68:32, or 87:13. Control septa had only hexane applied to them. Septa were stored inside glass vials at −20 °C when not in use.

### Behavioral bioassays

To avoid issues of contamination a clean Y-plate was used for every set of 15 or fewer replicates. Clean filter paper cut into the shape of the Y was inserted into the Y-plate to provide beetles with traction, and was discarded after each set. At the onset of each session, tests commenced by first offering beetles a choice in the Y-plate containing no odors (controls on both sides) in order to ensure that there was no bias in the apparatus due to lighting, airflow, contamination, or other factors. Once control beetles showed no bias, the lures were placed into the nozzles as described above and beetles were given a choice between a clean septum and an odor-laden septum. Beetles were individually placed into the Y through the hole at the bottom using a paint brush and allowed three minutes to make a choice. Beetles that entered one of the two arms and traveled at least half of the remaining distance from the junction to either side were scored as having made a choice. All other beetles were scored as non-responders. Once a beetle made a choice, that trial ended. The side of the Y-plate used to test the volatiles was alternated, and plates were cleaned thoroughly between changing sides or compounds. Behavioral testing was conducted between 1,030–1,530 h, under ambient fluorescent lighting, at 17–25 °C.

Using the Y-plate bioassays, a dose response test of the synthetic PSHB blend was conducted with mature female PSHB to evaluate their response to the two synthetic ketones, as well as to determine their optimal dose. Subsequently, the optimal dose was used to test mature females of each species for attraction to the two synthetic ketones at the three different ratios. Males of all three species were tested in Y-plate bioassays to determine their response to the blends as well. Finally, a one-eighth rubber septum containing 363 ng of quercivorol was tested with all three species to demonstrate attraction to a positive control, since some blends did not result in attraction.

### Statistical analysis

Pheromone component ratios for the three species were compared by dividing the amount of 2-21:Kt by the amount of 2-23:Kt in each extract of groups of beetles. After verifying equal variances, ratios were analyzed using ANOVA and Tukey means separations (*α* = 0.05) (JMP 10.0.0; SAS Institute, Inc., Cary, NC, USA).

Dual choice bioassays conducted in the Y-plate olfactometer were used to test the null hypothesis that both stimuli were chosen at the same frequency of 0.5. Because the requirements for using a Chi Square Goodness-of-fit test were frequently violated when fewer than five beetles selected one side (Sokal & Rohlf, 1995), the non-parametric two-tailed sign test was used to test the null hypothesis and significance level was determined using Statistical Table Q (Rohlf & Sokal, 1995).

## Results

### Qualitative analysis of PSHB samples

**Figure 1:**
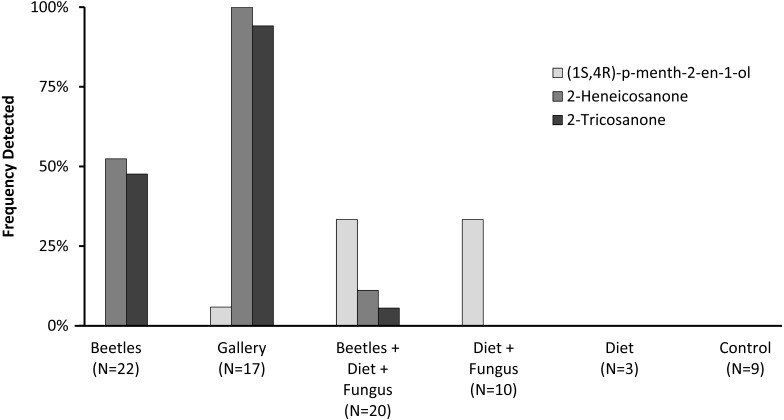
Frequency detected in volatile collections. Frequency (percent of samples) in which compounds were detected in volatile collections exploring for presence of potential pheromones. Samples were collected from different combinations of beetles, fungus, and diet, as listed on the *x*-axis. *N* indicates the number of volatile collections made and analyzed in each treatment.

**Figure 2:**
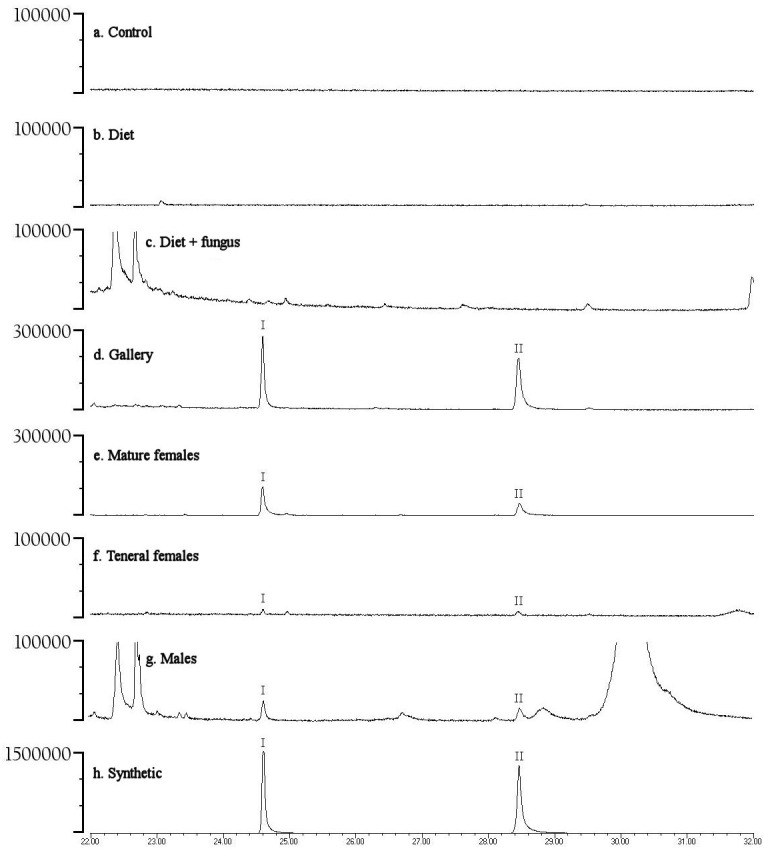
Representative gas chromatograms for volatiles collected from different treatments. GC traces showing volatiles from different PSHB treatments and controls eluting between 22 and 32 min. Note the *Y*-axes differ in abundance. Labeled compounds are 2-heneicosanone (I) and 2-tricosanone (II). PSHB, polyphagous shot hole borer (*E. sp #1*), the population from Los Angeles Co. (A) SPME fiber exposed in a control Pasteur pipette with glass wool for 120 min; (B) SPME fiber exposed to boxelder diet only (no fungus) inside of a Pasteur pipette for 960 min; (C) SPME fiber exposed to boxelder diet and fungus (non-gallery) from a PSHB colony tube inside of a Pasteur pipette for 75 min; (D) SPME inserted into gallery from the same PSHB colony tube for 1.5 min; (E) SPME fiber exposed to eleven mature female PSHB in a Pasteur pipette with glass wool for 60 min; (F) SPME fiber exposed to seven virgin teneral PSHB females in a Pasteur pipette with glass wool for 40 min; (G) 1 µl of extract of six PSHB males soaked in hexane for 2 d; (H) 50 ng each of synthetic 2-heneicosanone (I) and 2-tricosanone (II).

Preliminary studies explored volatiles from combinations of diet, fungus, and beetles. Using PSHB colonies, this exploratory phase revealed two ketones found only in the presence of beetles: 2-heneicosanone (2-21:Kt) and 2-tricosanone (2-23:Kt) (Fig. 1). Of 18 SHB galleries sampled with SPME fibers, the ketones were detected in all except one. Upon dissection of those rearing tubes, the gallery that did not contain either ketone was from a tube that had no live beetles present, and was thus reclassified into the “diet + fungus” treatment. The two ketones were also not found in any samples containing diet + fungus taken from the non-gallery parts of rearing tubes that contained active colonies, or diet + fungus from rearing tubes without live beetles. These two ketones were, however, isolated from both the headspace and extracts of beetles alone. The ketones were found in samples from mature adult females as well as virgin teneral females (Fig. 2). These two ketones were also discovered in small amounts in volatiles from males (Fig. 2G). Whole body extracts contained compounds also found in diet + fungus, as well as large GC peaks that appear to be cuticular hydrocarbons (Fig. 2G).

Quercivorol was found to be associated with volatile samples containing fungus, but was not detected in samples from diet or beetles in the absence of fungal growth. Quercivorol was found in both galleries and non-gallery samples of diet + fungus, both in the presence or absence of beetles (Fig. 1).

### Qualitative analysis of TSHB samples

Analysis of non-gallery diet and fungus, gallery, and males of the single TSHB colony revealed a similar pattern to that seen in PSHB. Headspace was sampled (SPME) from four treatments of a single 9-wk old TSHB rearing tube. The treatments were: diet + fungus, diet + fungus + males, females, and gallery. The two ketones, 2-21:Kt and 2-23:Kt, were found in all of these treatments except for the diet + fungus. It was noted that the ratio of the two ketones appeared to be different from that of PSHB based on peak area, which led to a quantitative examination of ratios for all three species.

**Table 1:** Amounts and ratios of the two ketone pheromone components in each species. Two hydrocarbon ketones were extracted from groups of beetles of each species and sex, and analyzed by GC-FID using an internal standard to quantify mean amount of each compound per beetle (ng ± SE). The mean ratios from mature females were subsequently used in behavioral bioassays.

		Females	Males
**PSHB**	**Mean ratio**	**45:55**	47:53
	2-21:Kt ng/beetle (mean ± SE)	40.9 ± 8.8	18.4
	2-23:Kt ng/beetle (mean ± SE)	50.0 ± 7.6	20.4
	N extractions	4	1
	Total no. of beetles extracted	81	10
**TSHB**	**Mean ratio**	**68:32**	71:29
	2-21:Kt ng/beetle (mean ± SE)	32.9 ± 1.2	13.3
	2-23:Kt ng/beetle (mean ± SE)	15.4 ± 0.2	5.4
	N extractions	2	1
	Total no. of beetles extracted	19	3
**KSHB**	**Mean ratio**	**87:13**	88:12
	2-21:Kt ng/beetle (mean ± SE)	65.3 ± 8.0	20.6
	2-23:Kt ng/beetle (mean ± SE)	10.2 ± 0.9	2.7
	N extractions	2	1
	Total no. of beetles extracted	31	5

**Notes.**

PSHB polyphagous shot hole borer from Los Angeles County, CA (E. sp. #1) TSHB tea shot hole borer from Miami-Dade County, FL (E. sp. #2) KSHB Kuroshio shot hole borer from San Diego County, CA (E. sp. #5) 2-21:Kt 2-heneicosanone 2-23:Kt 2-tricosanone

### Quantitative analysis of beetle-associated volatiles

**Figure 3:**
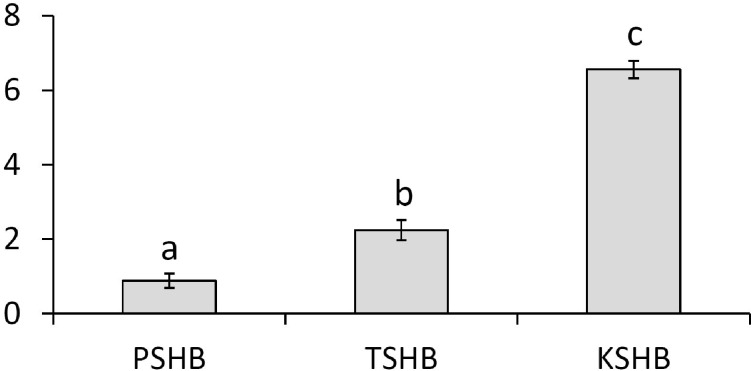
Quantitative comparison of pheromone component ratios between each species. Mean ratios (±SE) of the two hydrocarbon ketones, 2-heneicosanone and 2-tricosanone, found in extracts of three members of the *E. fornicatus* species complex invasive in the US. Beetles were extracted in pentane for 30 min and 2-tridecanone (internal standard) was added. *N* = 6, 3, and 4, extractions of groups of PSHB, TSHB, and KSHB beetles, respectively. Letters indicate significant differences (ANOVA and Tukey means separation *F* = 179.93, *P* < 0.0001, *α* = 0.05). PSHB, polyphagous shot hole borer, the population from Los Angeles Co. TSHB, tea shot hole borer, the population from Miami Dade Co. KSHB, Kuroshio shot hole borer, the population from San Diego Co. 2:21-Kt, 2-heneicosanone. 2:23-Kt, 2-tricosanone.

Extracts of male or female groups of PSHB, TSHB, and KSHB conducted with an internal standard revealed that the two ketones, 2-21:Kt and 2-23:Kt, were found at three different ratios among the three species, regardless of sex (Table 1, Fig. 3). Mean ratios of 2-21:Kt and 2-23:Kt in mature females were 45:55, 68:32, and 87:13 in PSHB, TSHB, and KSHB, respectively. They were also present at similar ratios in teneral adult females. When 2-21:Kt and 2-23:Kt were combined, mature female PSHB, TSHB, and KSHB produced about 91, 48, and 75 ng/beetle, respectively. Females produced more than twice as much of these two compounds than males (Table 1). Ratios of the two components differed significantly between the three species (Fig. 3) (ANOVA and Tukey means separation, *df* = 2, *F* = 179.93, *P* < 0.0001, *α* = 0.05).

### Behavioral bioassays

**Figure 4:**
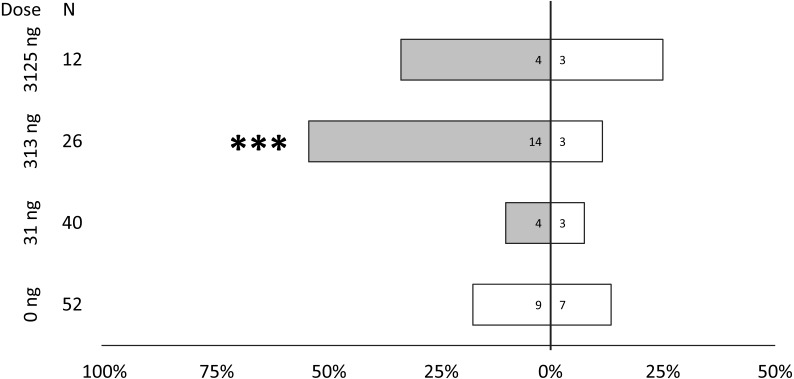
Dose response testing of synthetic pheromone blend. Walking responses of female PSHB in a Y-plate behavioral bioassay to three concentrations of the 45:55 blend of 2-heneicosanone and 2-tricosanone. Bars represent proportion of beetles making choices towards and away from the volatile source with respect to *N* beetles tested, using 1/8 rubber septa loaded with the compounds (shaded bars) or solvent controls (white bars). Numbers inside bars represent number of female beetles making each choice. Asterisks indicate significant difference from 50:50 (Sign test; *** *α* = 0.02).

Behavioral bioassays were conducted with female PSHB in a dose–response test to the synthetic blend of 2-21:Kt and 2-23:Kt at a ratio of 45:55, and doses of 0, 31, 313, and 3,125 ng. PSHB females responded dose-dependently with significant attraction to the 313 ng dose (Fig. 4).

**Figure 5:**
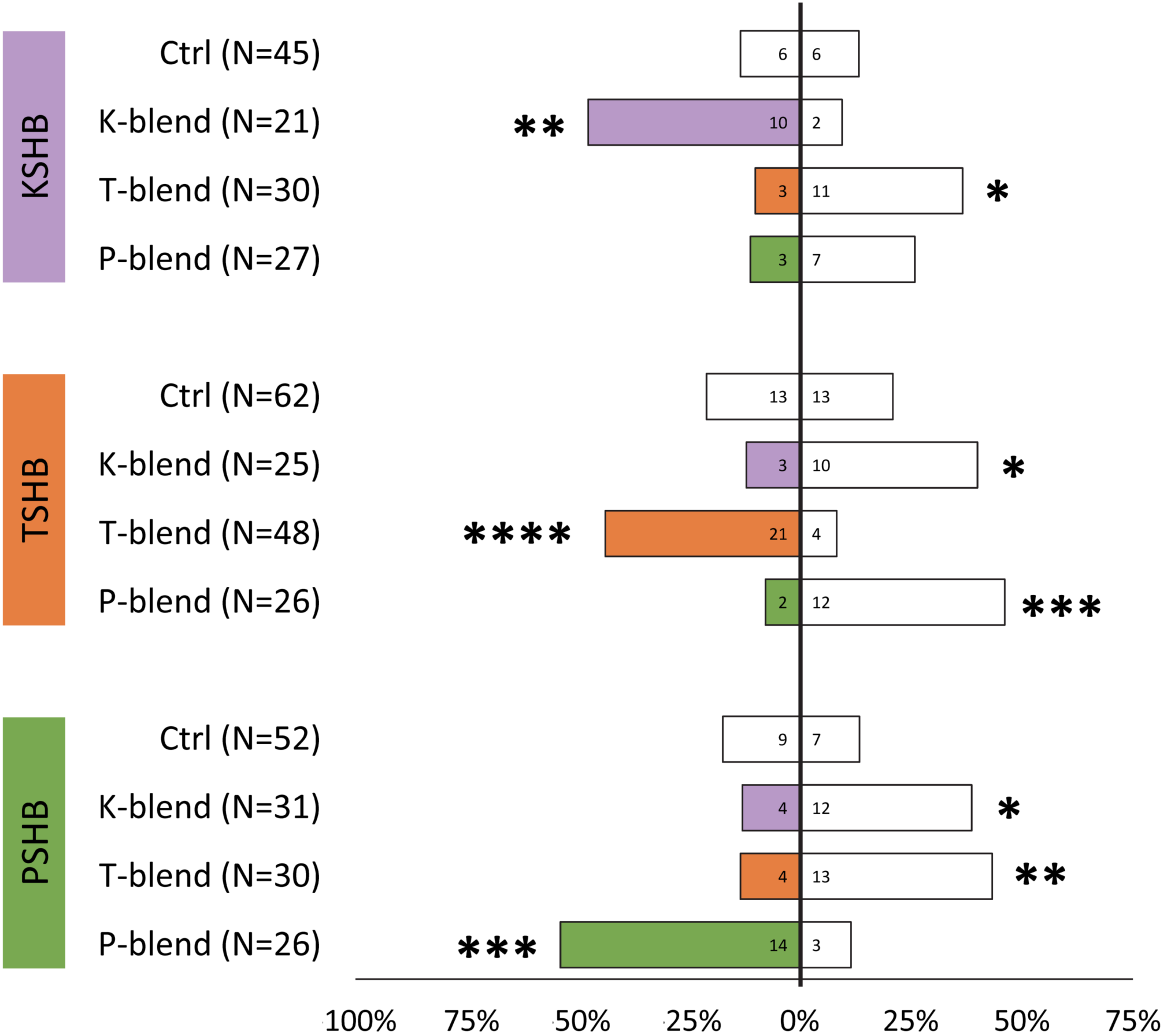
Female walking responses to synthetic pheromone components at different ratios. Female walking responses of the three *Euwallacea* species (KSHB, TSHB, and PSHB) in a Y-plate behavioral bioassay to three ratios of the two ketone pheromone components, 2-heneicosanone and 2-tricosanone, corresponding to the three species (K, T, P). Beetles were offered a choice between 313 ng of a synthetic pheromone blend (positive choice) and no odor (negative choice). Bars represent the proportion of female beetles making choices towards (shaded bars) and away from (white bars) the odor source with respect to *N* beetles tested. Numbers inside bars represent number of female beetles making each choice. Asterisks indicate significant difference from 50:50 (Sign test, **** *α* = 0.01; *** *α* = 0.02; ** *α* = 0.05; * *α* = 0.1). PSHB, polyphagous shot hole borer, the population from Los Angeles Co. TSHB, tea shot hole borer, the population from Miami Dade Co. KSHB, Kuroshio shot hole borer, the population from San Diego Co.

**Figure 6:**
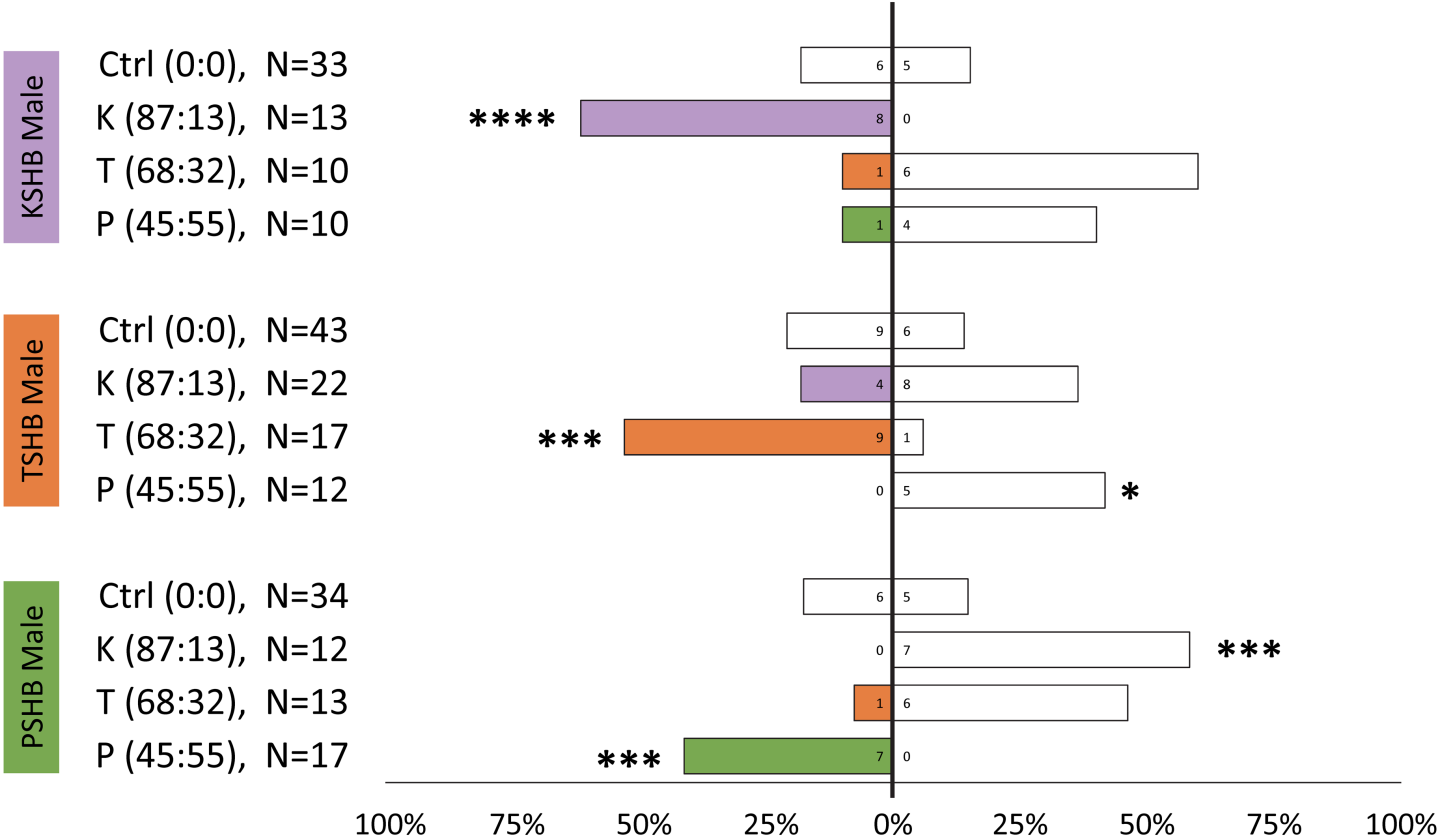
Male walking responses to synthetic pheromone components at different ratios. Male walking responses of the three *Euwallacea* species (KSHB, TSHB, and PSHB) in a Y-plate behavioral bioassay to three ratios of the two ketone pheromone components, 2-heneicosanone and 2-tricosanone, corresponding to the three species (K, T, P). Beetles were offered a choice between 313 ng of a synthetic pheromone blend (positive choice) and no odor (negative choice). Bars represent the proportion of male beetles making choices towards (shaded bars) and away from (white bars) the odor source with respect to *N* beetles tested. Numbers inside bars represent number of male beetles making each choice. Asterisks indicate significant difference from 50:50 (Sign test, **** *α* = 0.01; *** *α* = 0.02; ** *α* = 0.05; * *α* = 0.1). PSHB, polyphagous shot hole borer, the population from Los Angeles Co. TSHB, tea shot hole borer, the population from Miami Dade Co. KSHB, Kuroshio shot hole borer, the population from San Diego Co.

Lures with the 313 ng dose were tested subsequently with females of the three species to compare walking responses to blends with ratios corresponding to their natural ratios and to assess cross-attraction. Each of the three species were significantly attracted to synthetic versions of their own blend ratio, and significantly repelled by the blend ratios of the other two species (Fig. 5). Males were similarly found to be attracted to their own synthetic blends but not to blends matching the other two species (Fig. 6). The limited availability of males due to the extremely female-biased sex ratio resulted in fewer replicates. However, significant conspecific attraction by males was observed in all three species, and the same pattern of repellency trends were observed.

**Figure 7:**
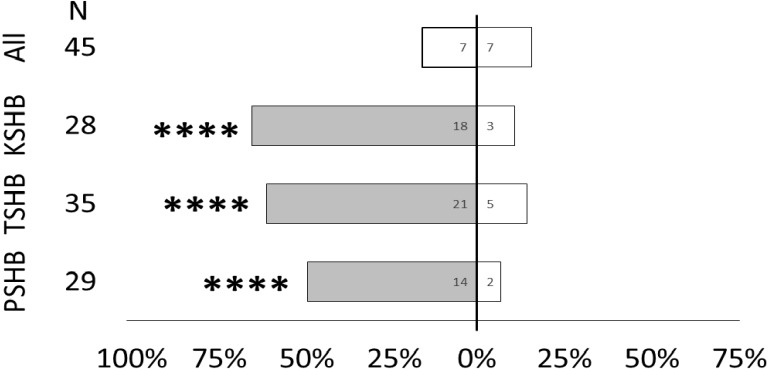
Responses of female PSHB, TSHB, and KSHB to quercivorol in the Y-plate bioassay. All three species of Euwallacea were attracted to a lure containing 363 ng of quercivorol in Y-plate behavioral bioassays. PSHB, polyphagous shot hole borer, the population from Los Angeles Co. TSHB, tea shot hole borer, the population from Miami Dade Co. KSHB, Kuroshio shot hole borer, the population from San Diego Co. All species were combined for testing the blank control. Bars represent proportion of female beetles making choices towards (shaded bars) and away from (white bars) the odor source with respect to *N* beetles tested. Numbers inside bars represent number of beetles making each choice. Asterisks indicate significant difference from 50:50 (Sign test, **** *α* = 0.01).

Quercivorol was found to be significantly attractive to mature females of all three species, and was used as a positive control to validate the walking assays (Fig. 7).

## Discussion

A variety of exploratory techniques revealed the presence of two ketones, 2-heneicosanone and 2-tricosanone associated with PSHB beetles. The most successful technique for demonstrating the presence or absence of these ketones in different treatments was by collecting head space volatiles with a SPME fiber inside a glass pipette which contained the volatile source. The systematic exploratory sampling of volatiles demonstrated that these two ketones were of beetle origin and not produced by the host plant material, diet, or symbiotic fungi. These ketones were found in both males and females (both teneral and mature females), and each of the three *Euwallacea* species were found to have the same two ketones, but at different ratios. Although ratios within a species were consistent for both males and females, females consistently produced more than two times the quantity of the two ketones than males. A dose-response test on the synthetic two-ketone blend, using the appropriate ratio for PSHB, demonstrated peak attraction by female PSHB beetles to the lure containing 313 ng of the blend. When the two ketones were tested in three ratios corresponding to the three *Euwallacea* species (PSHB, TSHB, and KSHB), females of each species were found to be significantly attracted to their own ratio, and significantly repelled by the other two ratios. Similarly, males were primarily attracted to their own ratio and not by ratios of the other species.

Although attraction to the pheromone blends was species-specific, quercivorol was found to be highly attractive to all three species. In volatile collections, quercivorol was not associated with beetles and was only detected in samples containing *Fusarium* fungus. Its presence only in samples containing fungus, and absence in samples containing only beetles or only diet, suggest that quercivorol is not a pheromone. That evidence as well as its attraction across all three species infers that quercivorol likely serves as a *Fusarium*-produced kairomone that is more broadly attractive to members of the *Euwallacea* species complex. Quercivorol has recently been demonstrated to attract all three species in the field (Carrillo et al., 2015; Dodge et al., 2017).

The chemical and behavioral data presented here indicates that these morphologically indistinguishable beetles, carrying different species of symbiotic *Fusarium*, and originating from three different invasive populations, have formed pheromone races, and further supports the amassing evidence that they have speciated into three cryptic species (Kasson et al., 2013; O’Donnell et al., 2015; Chen et al., 2016; Stouthamer et al., 2017). According to Stouthamer et al. (2017), multiple haplotypes were found of PSHB in Vietnam and Taiwan, of KSHB in Taiwan and Okinawa, and of TSHB in Thailand and India, suggesting their possible native origins. All three species were found to co-occur in Taiwan, PSHB and KSHB co-occured in Okinawa, and PSHB and TSHB co-occurred in Thailand, however populations represented by only one haplotype may represent invasions from other areas. PSHB and KSHB are less genetically divergent from each other than from TSHB (O’Donnell et al., 2015; Stouthamer et al., 2017), however, their pheromone ratios are the most divergent from each other, with the TSHB pheromone ratio being intermediate. The fact that PSHB and KSHB naturally coexist may have helped to drive the strong divergence of their pheromone component ratios, which could help avoid outbreeding. Since many ambrosia beetles are haplo-diploid, inbreed as a rule, and exhibit outbreeding depression (Haack, 2001; Peer & Taborsky, 2005), the resulting selective pressure would promote traits that reduce outbreeding, and could result in the evolution of divergent pheromones.

Although these pheromones may aid in avoidance of congeneric species, their ecological role and function is not yet understood, and there are several hypotheses for the possible ecological role they play. With respect to other pheromones, 2-heneicosanone and 2-tricosanone are relatively large molecules with lower volatility than most long distance aggregation or sex pheromones. Our preliminary field tests conducted in California to trap PSHB using their pheromone blend did not produce long range attraction. It is likely that these compounds are more akin to some insect trail pheromones such as (*Z*)-11-eicosesnal in the arboreal ant *Dolichoderus thoracicus* (Morgan, 2009) or (*Z*)-9-tricosene in the longhorn beetle *Anoplophora glabripennis* (Hoover et al., 2014). Since these two ketones were found in male beetles as well as both virgin and mated female beetles, and mated females were attracted to them, it is doubtful they act as sex pheromones. Aggregation is a possible function, but their high abundance inside galleries and their low volatility suggests they may function as trail pheromones, gallery-recognition pheromones, or pheromones to facilitate communication with nest mates, and might contribute to social behaviors such as cooperative brood care, as xyleborine beetles are predisposed for sociality (Biedermann, Klepzig & Taborsky, 2009). A mechanism to avoid congeners could be advantageous given that all three species share the highly attractive fungal kairomone quercivorol, whereas they likely face outbreeding depression (Peer & Taborsky, 2005) and appear to be obligate feeders on their own *Fusarium* species (Freeman et al., 2013a). Although beetles responded to their pheromone blends while walking upwind in a dual-choice olfactometer, we have not demonstrated attraction to them from a distance in the field. Testing for upwind flight response and close-range functions are topics for future investigation.

Although a number of pheromones are known for scolytine bark beetles, only a few examples of pheromones exist in scolytine ambrosia beetles, such as species in *Gnathotrichus* (Borden et al., 1976), *Trypodendron* (Borden & Slater, 1969), and *Xyletorus* (Francke & Heemann, 1974). *Gnathotrichus sulcatus* uses S-(+) and R-(−) sulcatol as an aggregation pheromone (Borden et al., 1976);, and *Trypodendron lineatum* (Coleoptera: Curculionidae) uses the aggregation pheromone lineatin during mass attack of new hosts (Borden & Slater, 1969; Borden, 1988). Pheromones have also been found in platypodid ambrosia beetles, such as the compounds (+)-sulcatol, sulcatone, and 3-pentanol used by males of *Megalyptus mutatus* (=*Platypus mutatus*) to attract females (Audino et al., 2005; Liguori et al., 2008), 1-hexanol, 3-methyl- 1-butanol, and sulcatol for aggregation by *Platypus flavicornis* (Renwick, Vité & Billing, 1977), and quercivorol reported as an aggregation pheromone for *Platypus quercivorus* (Kashiwagi et al., 2006).

This may be the first report of pheromones in the genus *Euwallacea,* or the use of 2-heneicosanone or 2-tricosanone in scolytine beetles. However, both of these methyl ketones have been reported in other arthropods. Interestingly, in social insects they occurred in the mandibular glands of stingless bees where they were proposed as possible constituents of a trail pheromone (Blum, 1970), and trace amounts of 2-tricosanone were found in the labial and tarsal glands of queen bumble bees *Bombus terrestris* (Hefetz, Taghizadeh & Francke, 1996). Both compounds were found in the cuticle of adult male and female pecan weevils *Curculio caryae* (Coleoptera: Curculionidae) (Espelie & Payne, 1991). The former compound, 2-heneicosanone, was found to occur on the tarsi and elytra of *Coccinella septempunctata* (Coleoptera: Coccinellidae), but was not found on tarsi or elytra of 34 other beetle species in eight other families (Geiselhardt, Geiselhardt & Peschke, 2011). In Lepidoptera, traces of these two ketones occurred among 66 compounds extracted from abdominal tips of male and mated female *Heliconius melpomene* butterflies, but not unmated females, leading to speculation that they may be part of a complex antiaphrodisiac pheromone blend (Schultz et al., 2008). Additionally, 2-heneicosanone was found in the hairpencils (male scent glands) of three species of African milkweed butterflies, *Amauris ochlea, A. damocles,* and *A. albimaculata* (Lepidoptera: Danaidae) (Schultz, Pobbré & Vane-Wright, 1993). Both ketones were also found in the cuticle and web of *Tegenaria atrica* spiders (Trabalon, Niogret & Legrand-Frossi, 2005). None of the above arthropod studies provided behavioral evidence of the roles of these ketones. Our study links these two compounds to species-specific ratios and behaviors and provides strong evidence that they are pheromones in three cryptic scolytine species.

Quercivorol has been documented as an attractant for the *E. fornicatus* species complex (Carrillo et al., 2015; Kendra et al., 2017; Dodge et al., 2017). It was first reported as an aggregation pheromone for the ambrosia beetle *Platypus quercivorus* (Coleoptera: Platypodidae) because it attracted both males and females (Tokoro et al., 2007). In that study, quercivorol was isolated from droplets excreted from the anus of fed virgin males, as well as from whole body extracts of fed males and newly emerged females of *P. quercivorus*, which unfortunately did not rule out the possibility of it originating in the ambrosia fungus *Raffaelea quercivora* and excreted in the feces. Our study found that for at least one member of the *Euwallacea fornicatus* species complex, quercivorol was associated with the ambrosia fungus and not the beetles, so in this system quercivorol seems to be a kairomone produced by their *Fusarium* ambrosia symbiont, rather than a pheromone. That these three cryptic species are each repelled by the pheromones of the other two, but are all attracted to quercivorol supports the notion of quercivorol as a kairomone in this system. Further investigation is needed on the cryptic members of the *E. fornicatus* species complex to understand the ecological role of the two ketones.

## Conclusions

In comparisons of volatiles from three cryptic species of the *E. fornicatus* species complex we found that beetles produced pheromones composed of two ketones: 2-heneicosanone and 2-tricosanone. These ketones were produced in unique ratios by each of the three species. When presented with synthetic blends of the ketones at the three ratios, beetles were attracted to their own ratio, and repelled by the ratios associated with the other two species. It is unlikely that these are sex pheromones or long range attractants being that these compounds are relatively high molecular weight and low volatility, both mated females and males produced them and were attracted to them, they were found in greatest abundance within the galleries, and the molecules are more akin to trail pheromones in other species. They may be involved in social behavior inside galleries or play a role in where foundresses initiate new colonies. Future work is needed to understand the full behavioral and ecological function of these pheromones.

##  Supplemental Information

10.7717/peerj.3957/supp-1 Table S1 Collecting PSHB volatiles using different approaches in search of possible pheromones The frequency that compounds were detected in collections (% of samples that were positive) are shown. Media are listed as either SPME fibers exposed to still air head space of the odor source, volatile collection of head space air flowing through a trap that was subsequently eluted into a solvent, or a direct solvent rinse or extract of the odor source. Odors were contained in and collected from various receptacles, consisting of either the rearing tube or a jar, a Pasteur pipette, within the beetle gallery in the colonized diet, or by touching the SPME fiber to the odor source.

10.7717/peerj.3957/supp-2 Table S2 Ketone ratio comparisons between species For each extraction of beetles in the quantitative analysis using an internal standard, the amount of each ketone (ng) per beetle was used to compare ratios between species.

10.7717/peerj.3957/supp-3 Figure S1 Diagram of the custom Y-plate bioassay design Y-plates used for bioassays were custom designed and cut from solid blocks of Teflon. Arrows indicate the direction of airflow. Disposable clear acetate sheets were sealed against the top and bottom of the plate with a bead of electrode gel. The nozzle tips were inserted snugly into the upwind ports pushing air in the direction of the arrows.
The single stem of the Y was 7.6 cm long, and two arms diverged at 90 degrees from each other. The two arms each had a 5.7 cm long section extending from the split, then a 45 degree bend which brought the final 1.8 cm sections parallel to each other. Each arm was 1.9 cm across. At each end of the two upper arms, a 0.635 cm hole was bored for the insertion of Teflon tubing (0.635 cm OD) for airflow into the bioassay.

10.7717/peerj.3957/supp-4 Figure S2 Nozzles for odor delivery in bioassays Nozzles were constructed using large and small pipette tips, respectively, cut (dotted lines) into parts (A) and (B). One eighth of a rubber septum (C) was placed into (B) which was placed into (A). Space can be seen between the two pipette tips (D) allowed clean air (dark arrows) to surround and mix with odor-laden air (light arrow). Bar measures 1 cm.
A preliminary attempt at odor delivery into the bioassay consisted of passing air through two flasks, one containing a lure and the other a control, and air from the two flasks was directed into the two arms of the Y. When visualized using smoke, it was found this produced a homogeneous odor plume on one half of the Y. However, this approach did not produce clear results as beetles responding to known attractants chose the control arm. It was suspected that the plume was too homogenous for beetles to navigate upwind in its center, and by navigating along the edge of clean air, they ended up in the wrong arm. In order to produce a heterogeneous plume composed of clean air interspersed with bursts of odors to allow optomotor anemotaxis to take place, custom nozzles were constructed which produced the desired effect and greatly improved the bioassay performance.
Air entered the two arms of the Y through a pair of custom nozzles crafted out of disposable pipette tips of two sizes, 1,000 µl and 100 µl (Finntip, Thermo Scientific, Waltham, MA). Both tips were cut (A, B). One eighth of a rubber septum was inserted into the smaller pipette tip, which was then inserted into the larger pipette tip (C). Ridges around the base of the smaller tip functioned as channels, allowing clean air to flow between the smaller tip and the larger tip (D). Air flowing through the inner tip flowed past the loaded septum and carried volatile compounds into the clean air stream which surrounded it. The tips of the nozzles were cut at an angle, the open side of which was directed toward the middle of the Y-plate. Nozzles were newly crafted for every set of tests and discarded afterwards.

10.7717/peerj.3957/supp-5 Data S1 List of volatile collections conducted in search of possible pheromones In exploratory volatile collections, for each sample the contents of the sample, approach used, and GCMS peak areas for the three compounds of interest are listed.

## Additional Information and Declarations

### Data Availability

The following information was supplied regarding data availability: The raw data for the behavioral bioassays is displayed in the figures within the manuscript, and is provided in the Supplemental Files.
