# Supplementary material for: Pheromones of three ambrosia beetles in the Euwallacea fornicatus species complex: ratios and preferences
Source: PeerJ. 2017 Oct 23;5:e3957. doi: 10.7717/peerj.3957 (PMC5657418; doi:10.7717/peerj.3957)
Supplement: Table S1 — The frequency that compounds were detected in collections (% of samples that were positive) are shown. Media are listed as either SPME fibers exposed to still air head space of the odor source, volatile collection of head space air flowing through a trap that was subsequently eluted into a solvent, or a direct solvent rinse or extract of the odor source. Odors were contained in and collected from various receptacles, consisting of either the rearing tube or a jar, a Pasteur pipette, within the beetle gallery in the colonized diet, or by touching the SPME fiber to the odor source. [file peerj-05-3957-s001.docx]

**SUPPLEMENTARY MATERIAL**

**Table 1**. Exploratory phase of collecting PSHB volatiles using different approaches. The frequency that compounds were detected in those collections (% of samples that were positive) are shown. Media are listed as either SPME fibers exposed to still air head space of the odor source, volatile collection of head space air flowing through a trap that was subsequently eluted into a solvent, or a direct solvent rinse or extract of the odor source. Odors were contained in and collected from various receptacles, consisting of either the rearing tube or a jar, a Pasteur pipette, within the beetle gallery in the colonized diet, or by touching the SPME fiber to the odor source.

| **Odor Source** | **Media** | **Receptacle** | **Avg. Time**  **(min.)** | **Frequency of Compound Detected by GCMS (% of samples positive)** | | | | **N** |  | |
| --- | --- | --- | --- | --- | --- | --- | --- | --- | --- | --- |
|  |  |  |  | **2-21:Kt** | **2-23:Kt** | **Quercivorol** |  |  | |  |
| **Beetles** |  |  |  |  |  |  |  |  | |  |
|  | SPME | Tube/Jar | 438 | 16.7 | 16.7 | 0 |  | 6 | |  |
|  | SPME | Pipette | 56 | 85.7 | 71.4 | 0 |  | 7 | |  |
|  | Rinse/extract | Tube/Jar | <1 | 100 | 66.7 | 0 |  | 3 | |  |
|  | Volatile collection | Tube/Jar | 1050 | 0 | 0 | 0 |  | 2 | |  |
|  | Volatile collection | Pipette | 5440 | 33.3 | 33.3 | 0 |  | 3 | |  |
| **Gallery** |  |  |  |  |  |  |  |  | |  |
|  | SPME | Pipette | 72 | 100 | 100 | 0 |  | 2 | |  |
|  | SPME | Gallery | 2 | 100 | 92.9 | 7.1 |  | 14 | |  |
|  | Rinse | Gallery | <1 | 100 | 100 | 0 |  | 1 | |  |
| **Diet + Fungus + Beetles** |  |  |  |  |  |  |  |  | |  |
|  | SPME | Tube/Jar | 19 | 0 | 0 | 16.7 |  | 6 | |  |
|  | SPME | Pipette | 90 | 25.0 | 25.0 | 75.0 |  | 4 | |  |
|  | SPME | Touch | <1 | 25.0 | 0 | 0 |  | 4 | |  |
|  | Volatile Collection | Tube/Jar | 670 | 0 | 0 | 50.0 |  | 4 | |  |
| **Diet + Fungus** |  |  |  |  |  |  |  |  | |  |
|  | SPME | Tube/Jar | 570 | 0 | 0 | 0 |  | 3 | |  |
|  | SPME | Gallery | <1 | 0 | 0 | 0 |  | 1 | |  |
|  | SPME | Pipette | 43 | 0 | 0 | 40.0 |  | 5 | |  |
|  | Volatile collection | Tube/Jar | 360 | 0 | 0 | 100 |  | 1 | |  |
| **Diet** |  |  |  |  |  |  |  |  | |  |
|  | SPME | Pipette | 595 | 0 | 0 | 0 |  | 3 | |  |
| **Control** |  |  |  |  |  |  |  |  | |  |
|  | SPME | Tube/Jar | 485 | 0 | 0 | 0 |  | 4 | |  |
|  | SPME | Pipette | 58 | 0 | 0 | 0 |  | 2 | |  |
|  | Volatile collection | Vial/Jar | 360 | 0 | 0 | 0 |  | 1 | |  |
|  | Volatile collection | Pipette | 3840 | 0 | 0 | 0 |  | 2 | |  |
