# Supplementary material for: Pheromones of three ambrosia beetles in the Euwallacea fornicatus species complex: ratios and preferences
Source: PeerJ. 2017 Oct 23;5:e3957. doi: 10.7717/peerj.3957 (PMC5657418; doi:10.7717/peerj.3957)
Supplement: Table S2 — For each extraction of beetles in the quantitative analysis using an internal standard, the amount of each ketone (ng) per beetle was used to compare ratios between species. [file peerj-05-3957-s002.docx]

**SUPPLEMENTARY MATERIAL**

**Table 2.** Raw data for ratio comparisons between species.

|  |  | **ng/beetle** | |  |
| --- | --- | --- | --- | --- |
| **Species** | **Beetles Extracted** | **2-21:Kt** | **2-23:Kt** | **Ratio (2-heneicosanone/2-tricosanone)** |
| PSHB | 15 mature females | 62.5 | 71.1 | 0.8790 |
|  | 18 mature females | 47.5 | 50.9 | 0.9332 |
|  | 17 mature females | 30.3 | 40.3 | 0.7518 |
|  | 31 mature females | 23.3 | 37.6 | 0.6197 |
|  | 14 teneral females | 49.9 | 41.1 | 1.2141 |
|  | 10 males | 18.4 | 20.4 | 0.9020 |
|  |  |  |  |  |
| TSHB | 10 mature females | 34 | 15.5 | 2.1935 |
|  | 9 mature females | 31.7 | 15.2 | 2.0855 |
|  | 3 males | 13.3 | 5.4 | 2.4630 |
|  |  |  |  |  |
| KSHB | 16 mature females | 73.2 | 11 | 6.6545 |
|  | 15 mature females | 57.3 | 9.3 | 6.1613 |
|  | 5 males | 20.6 | 2.7 | 7.6296 |
|  | 10 teneral females | 52 | 9 | 5.7778 |
