# Supplementary figures and images for: Pheromones of three ambrosia beetles in the Euwallacea fornicatus species complex: ratios and preferences
Source: PeerJ. 2017 Oct 23;5:e3957. doi: 10.7717/peerj.3957 (PMC5657418; doi:10.7717/peerj.3957)

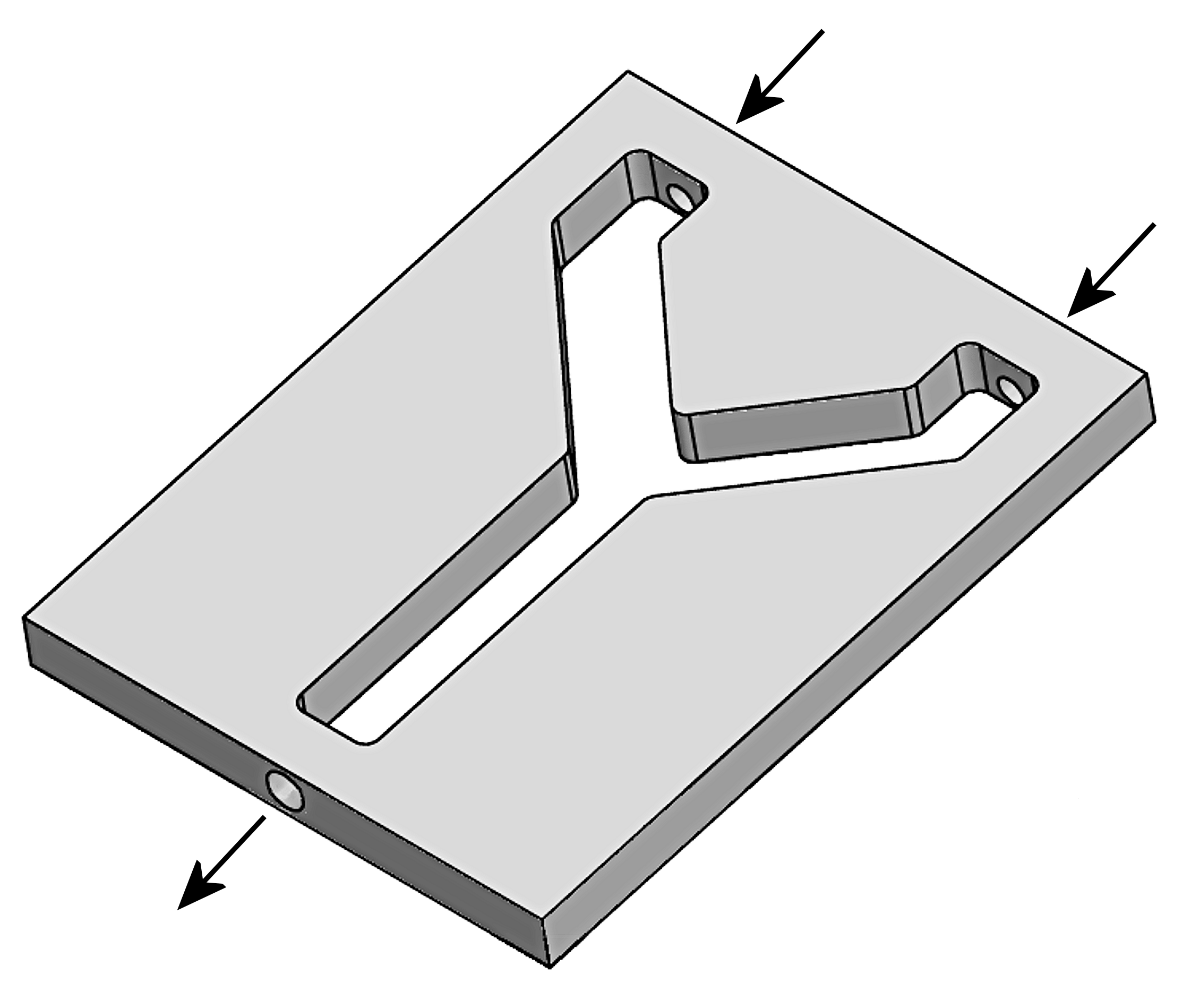

Supplement: Figure S1 — Y-plates used for bioassays were custom designed and cut from solid blocks of Teflon. Arrows indicate the direction of airflow. Disposable clear acetate sheets were sealed against the top and bottom of the plate with a bead of electrode gel. The nozzle tips were inserted snugly into the upwind ports pushing air in the direction of the arrows. The single stem of the Y was 7.6 cm long, and two arms diverged at 90 degrees from each other. The two arms each had a 5.7 cm long section extending from the split, then a 45 degree bend which brought the final 1.8 cm sections parallel to each other. Each arm was 1.9 cm across. At each end of the two upper arms, a 0.635 cm hole was bored for the insertion of Teflon tubing (0.635 cm OD) for airflow into the bioassay. [file peerj-05-3957-s003.png]

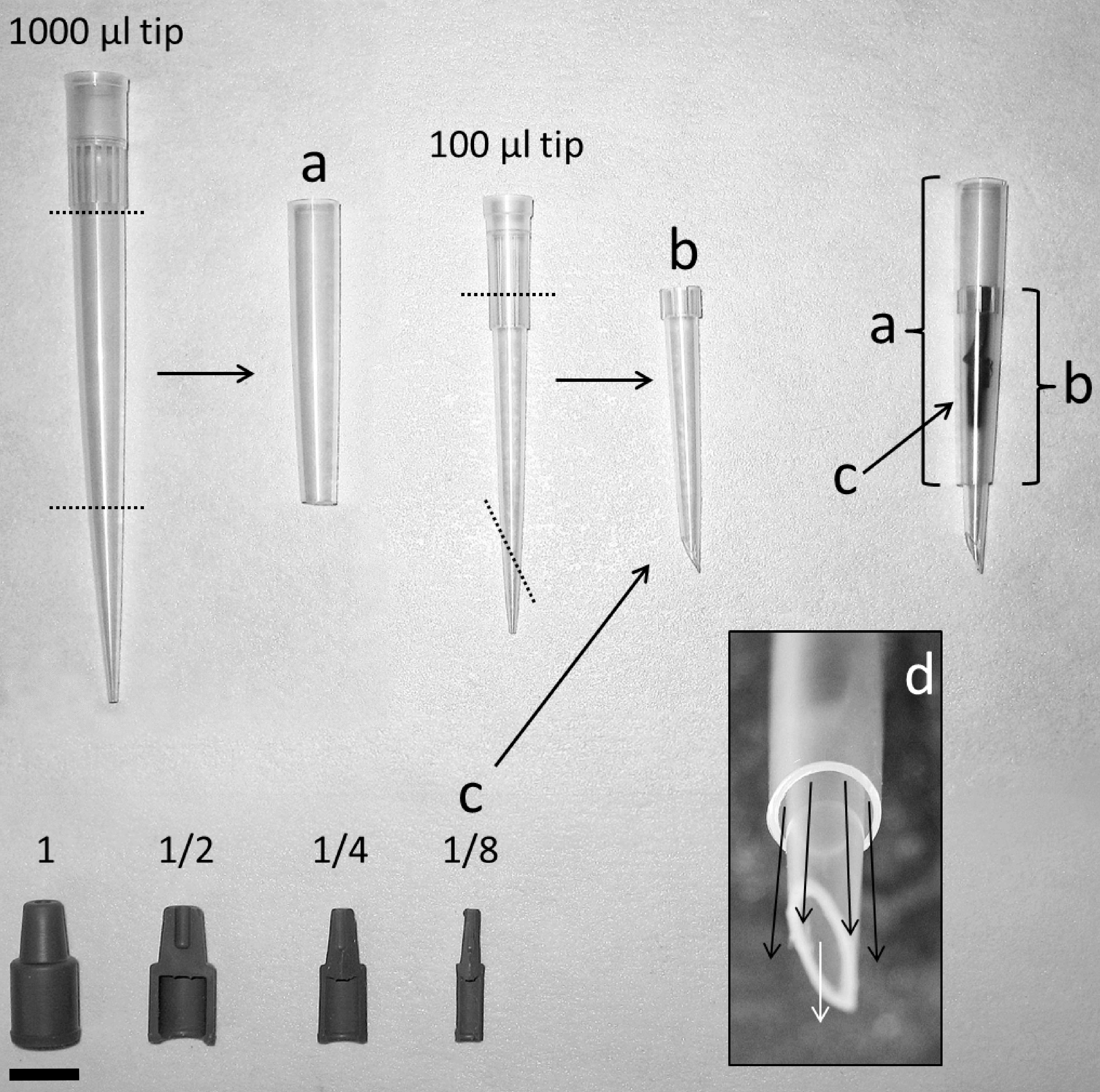

Supplement: Figure S2 — Nozzles were constructed using large and small pipette tips, respectively, cut (dotted lines) into parts (A) and (B). One eighth of a rubber septum (C) was placed into (B) which was placed into (A). Space can be seen between the two pipette tips (D) allowed clean air (dark arrows) to surround and mix with odor-laden air (light arrow). Bar measures 1 cm. A preliminary attempt at odor delivery into the bioassay consisted of passing air through two flasks, one containing a lure and the other a control, and air from the two flasks was directed into the two arms of the Y. When visualized using smoke, it was found this produced a homogeneous odor plume on one half of the Y. However, this approach did not produce clear results as beetles responding to known attractants chose the control arm. It was suspected that the plume was too homogenous for beetles to navigate upwind in its center, and by navigating along the edge of clean air, they ended up in the wrong arm. In order to produce a heterogeneous plume composed of clean air interspersed with bursts of odors to allow optomotor anemotaxis to take place, custom nozzles were constructed which produced the desired effect and greatly improved the bioassay performance. Air entered the two arms of the Y through a pair of custom nozzles crafted out of disposable pipette tips of two sizes, 1,000 µl and 100 µl (Finntip, Thermo Scientific, Waltham, MA). Both tips were cut (A, B). One eighth of a rubber septum was inserted into the smaller pipette tip, which was then inserted into the larger pipette tip (C). Ridges around the base of the smaller tip functioned as channels, allowing clean air to flow between the smaller tip and the larger tip (D). Air flowing through the inner tip flowed past the loaded septum and carried volatile compounds into the clean air stream which surrounded it. The tips of the nozzles were cut at an angle, the open side of which was directed toward the middle of the Y-plate. Nozzles were newly crafted for ev [file peerj-05-3957-s004.png]
